# A multi-analyte blood test for acute spinal cord injury

**DOI:** 10.1172/JCI185463

**Published:** 2025-03-03

**Authors:** Tej D. Azad, Kathleen R. Ran, Joshua D. Materi, Divyaansh Raj, Timour Al-Khindi, Sameer Gabbita, Marvin Li, Elizabeth T. Wang, A. Karim Ahmed, Megan Parker, Anita L. Kalluri, Daniel Lubelski, Christopher M. Jackson, Daniel M. Sciubba, Jon D. Weingart, Ali Bydon, Timothy F. Witham, David W. Nauen, Srinivasan Yegnasubramanian, Nicholas Theodore, Chetan Bettegowda

**Affiliations:** 1 Department of Neurosurgery, Johns Hopkins University School of Medicine, Baltimore, Maryland, USA.; 2 Department of Neurosurgery, Zucker School of Medicine at Hofstra, Manhasset, New York, USA.; 3 Department of Pathology and; 4 Department of Oncology, Johns Hopkins University School of Medicine, Baltimore, Maryland, USA.

**Keywords:** Neuroscience, Neurological disorders

## Abstract

**BACKGROUND:**

Rapid diagnosis to facilitate urgent intervention is critical for treatment of acute spinal cord injury (SCI). We hypothesized that a multi-analyte blood biomarker would support point-of-care SCI diagnosis, correlate with injury severity, and predict long-term neurologic outcomes.

**METHODS:**

Droplet digital PCR (ddPCR) assays were designed to amplify differentially hypomethylated genomic loci in spinal cord tissue. An optimized ddPCR assay was applied to cell-free DNA (cfDNA) from plasma samples collected from prospectively enrolled acute SCI patients. Targeted proteomic profiling was also performed. Spinal cord–derived cfDNA and plasma proteins were tested for their association with SCI and ability to predict conversion in American Spinal Injury Association (ASIA) score at 6 months.

**RESULTS:**

A bespoke ddPCR assay detected spinal cord–derived cfDNA in plasma of 50 patients with acute SCI (AUC: 0.89, 95% CI 0.83–0.95, *P* < 0.0001). Levels of cfDNA were highest in patients with the most severe injury, i.e., ASIA A, compared with those with ASIA B (*P* = 0.04), ASIA C (*P* = 0.009), and ASIA D injuries (*P* < 0.001). Dimensionality reduction identified 4 candidate proteins (FABP3, REST, IL-6, NF-H) that were integrated with spinal cord–derived cfDNA to derive the Spinal Cord Injury Index (SCII), which has high sensitivity and specificity for SCI diagnosis (AUC: 0.91, 95% CI 0.82–0.99, *P* < 0.0001), correlates with injury severity (*P* < 0.0001), and predicts 6-month neurologic improvement (AUC: 0.77, 95% CI 0.61–0.93, *P* = 0.006).

**CONCLUSION:**

The detection of spinal cord–derived cfDNA and plasma protein alterations as part of a multi-analyte blood test can inform SCI diagnosis and prognosis.

**FUNDING:**

North American Spine Society Young Investigator Award; Morton Cure Paralysis Fund.

## Abstract 2

We developed a multi-analyte blood test for acute spinal cord injury to enable prompt diagnosis, quantfication of injury severity, and prediction of neurologic outcomes.

## Introduction

Acute spinal cord injury (SCI) results in lasting neurological deficits, decreased life expectancy, and immense psychosocial tolls on patients and caregivers (1–3). SCI also results in substantial socioeconomic burden; in the United States alone, SCI is estimated to cost more than $9.7 billion annually (2). Prompt diagnosis with physical examination and advanced neuroimaging followed by urgent surgical decompression of the spinal cord is considered standard of care for optimizing long-term neurologic function by limiting secondary injury (4–6). Adjuvant therapies, such as hemodynamic augmentation, modulation of intraspinal pressure, and neuroprotective agents, remain under investigation (7–11). Currently, there is no clinically useful biomarker to expedite SCI diagnosis, quantify injury severity, predict therapeutic response, facilitate clinical trials, or enable effective prognostication (12).

Given this unmet clinical need, both cerebrospinal fluid (CSF) and peripheral blood biomarkers of SCI have been investigated. Putative CSF biomarkers include metabolites, microRNAs, and neuroglial proteins (13–15). Metabolomic and microRNA profiling have demonstrated greater utility in CSF, while protein biomarkers may achieve comparable performance in blood and CSF (15). Given the ease of obtaining peripheral blood compared with CSF, blood-based SCI biomarkers are preferable.

**Figure 1:**
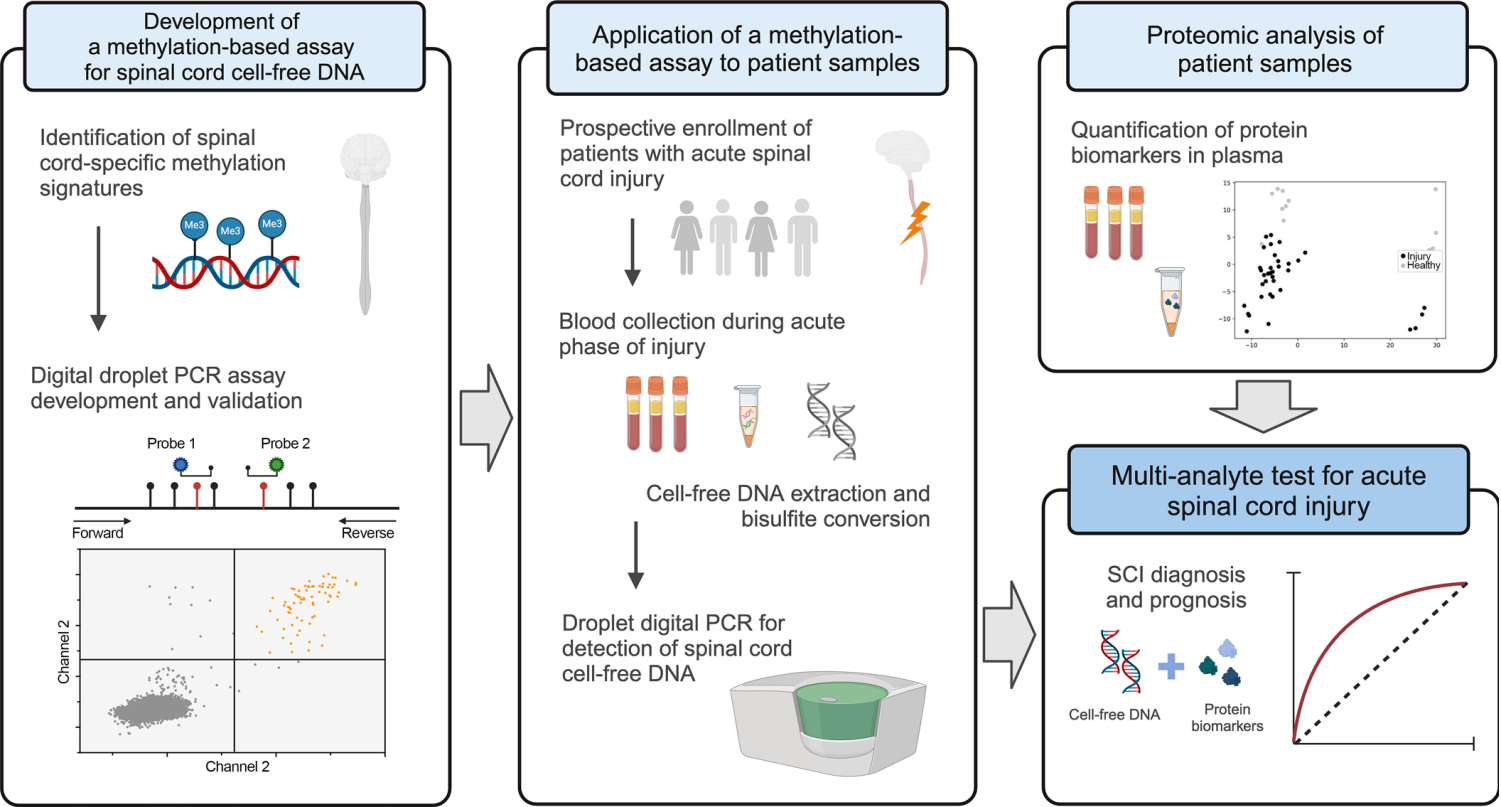
Overview of study for detection of acute SCI via a multi-analyte assay of peripheral blood.

Recently, detection of tissue-specific cell injury using conserved methylation patterns on cell-free DNA (cfDNA) led to the discovery of circulating biomarkers for myocardial infarction, acute rejection after liver transplant, and graft-versus-host disease (16–18). We sought to translate this framework to the central nervous system (CNS), hypothesizing that spinal cord–derived methylation patterns in cfDNA may form the basis of blood biomarkers for SCI. Drawing from advances in cancer biomarker development, where cfDNA has been integrated with proteins, we hypothesize that a multi-analyte approach to SCI biomarker discovery may realize orthogonal contributions of cfDNA and circulating proteins (Figure 1) (19, 20).

## Results

### Cohort overview.

**Figure 2:**
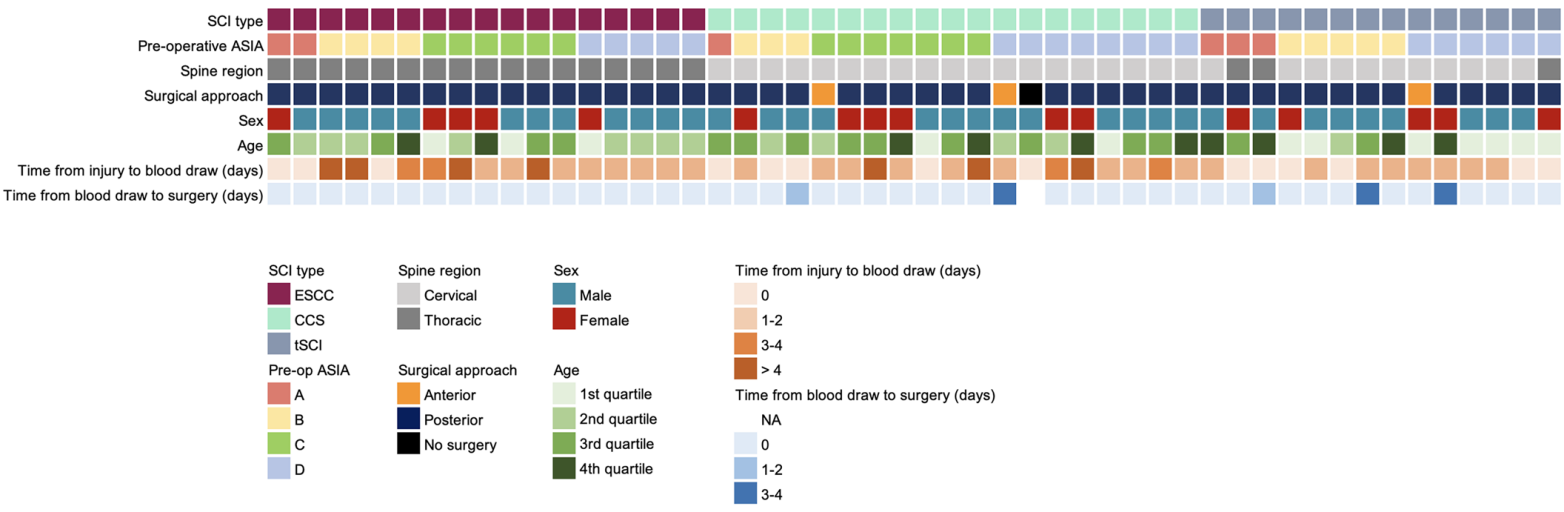
Cohort overview with patient, operative, and injury characteristics. NA, not applicable.

We prospectively enrolled 50 patients with acute SCI at 2 institutions (Figure 2 and Supplemental Table 1; supplemental material available online with this article; https://doi.org/10.1172/JCI185463DS1). SCI types included traumatic SCI (tSCI) (*n* = 14), traumatic central cord syndrome (CCS) (*n* = 19), and acute epidural spinal cord compression (ESCC) (*n* = 17). Presenting neurologic status (American Spinal Injury Association [ASIA] Impairment Scale [AIS]) was A in 12%, B in 24%, C in 26%, and D in 38%. Most patients were male (68%), sustained a cervical level injury (60%), and underwent surgery via a posterior approach (94%) (21).

### Identification of spinal cord–specific methylation loci.

We performed Illumina MethylationEPIC 850K array-based methylation profiling on fresh, frozen human spinal cord tissue obtained from an institutional rapid autopsy program (*n* = 3 unique patients) and on formalin-fixed, paraffin-embedded (FFPE) human spinal cord tissue (*n* = 3 unique patients) without spinal cord pathology. We compared the results in silico from these 6 samples to methylation profiles from 25 other tissue and cell types, including cultured human cortical neurons, to identify CpG sites differentially methylated in spinal cord tissue.

**Figure 3:**
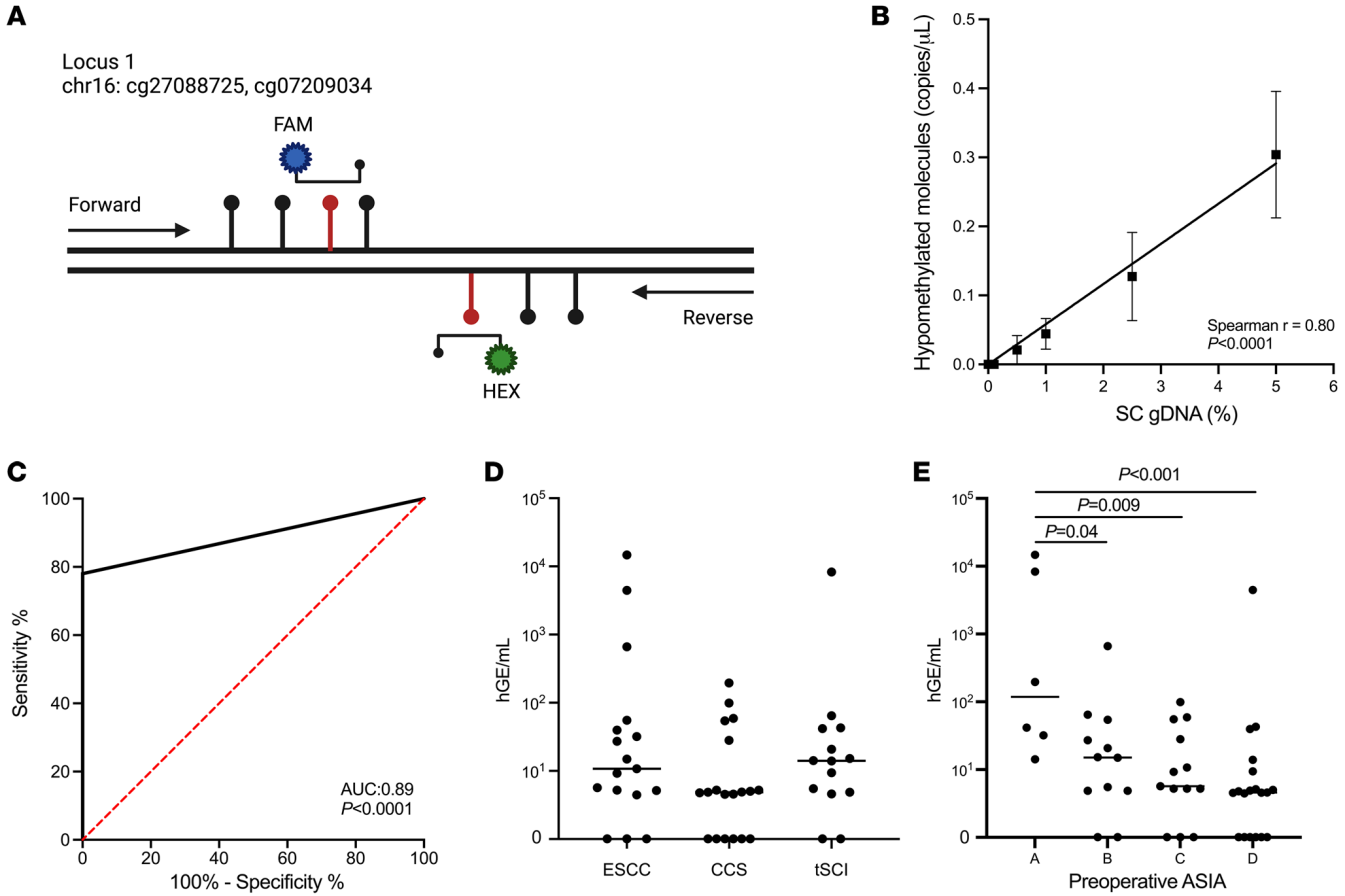
Methylation-based cfDNA markers of SCI. (**A**) Structure at a genomic locus identified as spinal cord–specific biomarker. Lollipops represent CpG sites. Red highlights hypomethylated CpG sites identified in the Illumina MethylationEPIC 850K array. Arrows mark positions of PCR primers. (**B**) Spike-in experiment demonstrating the ddPCR assay sensitivity for detection of spinal cord–derived biomarkers. Human spinal cord gDNA was mixed with human leukocyte gDNA in the indicated proportions (0 to 5%), and the concentration (copies/uL) of fully unmethylated spinal cord–derived markers was determined. Data represent mean ± SEM. (**C**) Receiver operating curve demonstrating the ability of our hypomethylation-based ddPCR assay to discriminate between acute SCI patients and healthy controls. (**D**) Plasma concentration in genome equivalents (hGE/mL) of spinal cord–derived cfDNA in acute SCI patients. (**E**) Percentage of spinal cord–derived cfDNA out of total measured cfDNA in acute SCI patients. Mann-Whitney *U* tests were used to compare groups in panel **E**. AUC, area under the receiver operating curve.

Two candidate regions were selected in silico based on having the greatest mean difference between spinal cord and all other tissue or cell types and containing multiple CpG sites that could be captured within a single cfDNA molecule. We designed bespoke droplet digital PCR (ddPCR) assays to amplify these regions if all included CpG sites were unmethylated (Figure 3A; assay 1: 2 CpG sites; assay 2: 3 CpG sites). When applied to bisulfite converted genomic DNA (gDNA) from human spinal cord tissue and PBMCs from non-SCI control patients, assay 1 demonstrated robust detection of spinal cord tissue gDNA (i.e., target CpG sites were hypomethylated) and no detection of spinal cord–specific cfDNA in non-SCI control cfDNA. (Supplemental Figure 1, A and B). We established a standard curve by spiking known concentrations of spinal cord gDNA into control PBMC gDNA, further demonstrating that assay 1 had markedly better calibration at low spinal cord gDNA concentrations and demonstrated linearity (assay 1: Spearman, ρ = 0.80, *P* < 0.0001, Figure 3B; assay 2: Spearman, ρ = 0.64, *P* < 0.01 Supplemental Figure 2). We further confirmed that assay 1 did not amplify when applied to acute SCI patient PBMC gDNA (Supplemental Figure 1, C and D). As a result, all further ddPCR analyses were performed utilizing assay 1. To conduct further technical validation, we designed a “reverse assay,” in which CpG sites of interest were inverted (i.e., assay designed to detect hypermethylation at target sites, rather than hypomethylation) and likely to represent non–spinal cord–derived DNA. As predicted, detection using the reverse assay was inversely correlated with the amount of spiked-in spinal cord gDNA (Supplemental Figure 3, A and B).

### Detection of spinal cord–derived cfDNA in peripheral blood.

We applied the optimized ddPCR assay to bisulfite converted cfDNA from plasma samples of 50 patients with acute SCI and 20 non-SCI control subjects. Median time from reported neurologic deficit or injury to blood draw was 1 day (interquartile range [IQR], 0–2 days) for SCI patients, and all draws were obtained prior to surgery. The absolute plasma concentration of spinal cord–derived cfDNA was measured in haploid genome equivalents per milliliter of plasma (hGE/mL). Spinal cord–specific cfDNA was detected in 39 of 50 patients with acute SCI and in 0 of 20 non-SCI control patients, yielding a sensitivity of 78% and a specificity of 100% for diagnosis of acute SCI (Figure 3C). The concentration of spinal cord–derived cfDNA did not significantly differ across SCI etiologies (Figure 3D) but did vary with SCI severity (Figure 3E). Significantly higher levels of cfDNA were measured in ASIA A injuries compared with ASIA B (*P* = 0.04), ASIA C (*P* = 0.009), and ASIA D injuries (*P* < 0.001). Median spinal cord–derived concentrations for ASIA A patients were 118.7 hGE/mL (IQR, 27.5–9907 hGE/mL), 15.1 hGE/mL (IQR, 4.87–47.6 hGE/mL) for ASIA B patients, 5.67 hGE/mL (IQR, 2.62–41.6 hGE/mL) for ASIA C patients, and 4.60 hGE/mL (IQR, 0–9.43 hGE/mL) for ASIA D patients.

### Proteomic profiling of plasma from SCI patients.

Next, we performed proteomic profiling of 119 CNS-related proteins using a recently established proximity ligation assay, nucleic acid linked immuno-sandwich assay (NULISA, Alamar Biosciences), optimized for circulating biomarker development (22). Proteomic profiling was performed in a subset of 34 acute SCI patients and 12 non-SCI controls. Importantly, the proteomic profiling was performed from the same physical sample from which cfDNA was extracted for all SCI patients.

**Figure 4:**
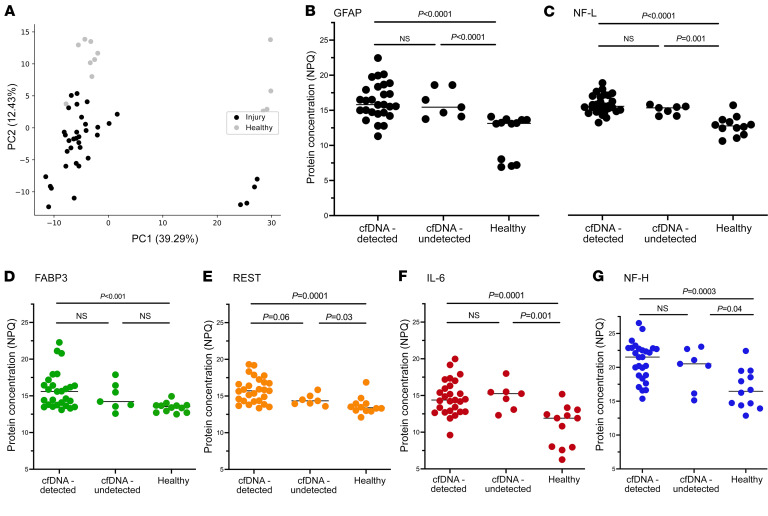
Proteomic profiling of plasma from patients with acute SCI. (**A**) PCA of protein biomarkers measured in acute SCI patients versus healthy controls. (**B** and **C**) Difference in GFAP and NF-L concentrations between acute SCI patients detected using ddPCR and healthy controls. (**D**–**G**) Difference in FABP3, REST, IL-6, and NF-H concentrations between acute SCI patients detected using ddPCR and healthy controls. Mann-Whitney *U* tests were used to compare groups in panels **B**–**G**.

Protein levels were normalized to an internal control, rescaled, log_2_ transformed, and represented as NULISA protein quantification (NPQ) units (further detail in Methods). Principal component analysis (PCA) demonstrated distinct separation of proteomic signatures between injured versus healthy subjects (Figure 4A). Previously, glial fibrillary acidic protein (GFAP) and neurofilament light (NF-L) were measured in serum and CSF of patients with tSCI and were found to be associated with injury severity and 6-month neurologic outcomes (15). Similarly, we found that GFAP and NF-L levels were significantly higher in SCI patients, relative to non-SCI controls (*P* < 0.001). However, we observed that GFAP and NF-L levels did not significantly differ between SCI patients in whom spinal cord–derived cfDNA was detected and SCI patients in whom spinal cord–derived cfDNA was not detected (Figure 4, B and C). Both GFAP (Spearman, ρ = 0.42, *P* = 0.004) and NF-L (Spearman, ρ = 0.70, *P* < 0.0001) levels significantly correlated with concentrations of spinal cord–derived cfDNA.

We then worked to identify parsimonious sets of proteins associated with the main clinical outcome of interest: 6-month ASIA conversion. To achieve this, we ranked proteins based on difference in median NPQ values between SCI patients who converted and those who did not. We then performed forward selection in a logistic regression model to identify a set of proteins associated with 6-month ASIA conversion. This approach identified 4 proteins: fatty acid binding protein 3 (FABP3), RE1-silencing transcription factor (REST), IL-6, and neurofilament heavy polypeptide (NF-H). Levels of all 4 proteins were significantly higher in SCI patients in whom spinal cord–derived cfDNA was detected, compared with non-SCI controls (all *P* < 0.001). Levels of REST, IL-6, and NF-H were higher in SCI patients without detection of spinal cord–derived cfDNA, relative to non-SCI controls (Figure 4, D–G).

### Development of the SCII.

**Figure 5:**
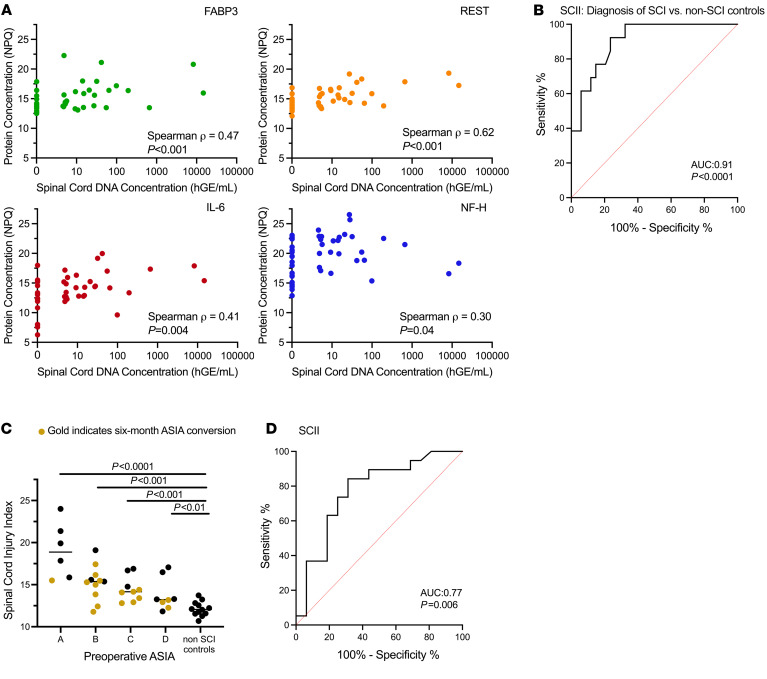
Development of the SCII. (**A**) Correlation between concentrations of spinal cord–derived cfDNA and FABP3, REST, IL-6, and NF-H. (**B**) Receiver operating characteristic (ROC) curve for ability of SCII to discriminate between patients with acute SCI and healthy controls. (**C**) Distribution of SCII across injury severity. Yellow indicates patient who achieved 6-month ASIA conversion. (**D**) Receiver operating characteristic curve for ability of preoperative SCII to predict 6-month ASIA conversion. Mann-Whitney *U* tests were used to compare groups in panel **C**.

Next, we assessed the association between concentration of spinal cord–derived cfDNA with these 4 proteins (Figure 5A). We found consistent positive correlations: FABP3 (Spearman, ρ = 0.47, *P* < 0.001), REST (Spearman, ρ = 0.62, *P* < 0.001), IL-6 (Spearman, ρ = 0.41, *P* = 0.004), and NF-H (Spearman, ρ = 0.30, *P* = 0.04).

To develop a composite score reflecting neuroglial injury after SCI, we linearly combined NPQ values of the 4 selected proteins using coefficients from the logistic regression used for candidate selection and the log_10_ scaled spinal cord–derived cfDNA concentration. For samples with undetectable cfDNA, we imputed the limit of detection estimated from standard curve experiments. This integrated value is summarized as the SCII. Though non-SCI controls were not used to inform SCII development, we observed that the SCII reliably distinguished acute SCI patients from non-SCI controls (AUC = 0.91, *P* < 0.0001) (Figure 5B). The SCII demonstrated a step-wise decrease with decreasing injury severity (Kruskal-Willis, *P* < 0.0001) with relatively tight clustering within AIS groups (Figure 5C). Finally, we sought to confirm that the SCII predicted 6-month ASIA conversion, observing that the SCII discriminated between SCI patients who converted (*n* = 18) and those who did not (*n* = 16) with an AUC of 0.77 (*P* = 0.006, Figure 5D). Importantly, the composite SCII score achieved greater discriminative performance of ASIA conversion at 6 months than either cfDNA or protein alone (Supplemental Figure 4, A–C).

## Discussion

No clinically useful biomarkers exist to facilitate earlier diagnosis or enable prognostication for patients with acute SCI. This represents a fundamental gap in the care of this population. Our investigation demonstrates that detection of spinal cord–derived cfDNA and plasma protein alterations as part of a multi-analyte blood test can inform SCI diagnosis and prognosis.

A blood biomarker presents several potential contributions to the clinical management of patients with SCI. First, it provides a minimally invasive and widely accessible means of diagnosis. Current means of diagnosing SCI are heavily dependent upon advanced neuroimaging studies, commonly MRI. However, MRI may not be readily available in resource-limited settings and may not be safe in patients with metal implants, severe claustrophobia, or other contraindications. In contrast, blood samples can be readily acquired via venipuncture routinely performed as part of standard clinical practice for patients with suspected SCI. There is also a need for more objective methods of assessing SCI at presentation and predicting outcomes. Currently, the ASIA grading system is commonly used to assess injury severity at presentation and prognosticate outcomes based on acute evaluation of sensory and motor function (21). However, accurate ASIA grading may be challenging or not feasible in SCI patients who are unresponsive, have cognitive disturbance, or have difficulty cooperating with the exam due to their acute injury (23). During the first 48 hours after injury, ASIA grading is frequently unstable due to spinal shock or sedation (24). Moreover, neurologic examination and neuroimaging have limited spatiotemporal resolution for capturing pathophysiologic changes, which may inform observed variability in rates of neurologic recovery (15, 24, 25). Additionally, there are no reliable methods for selecting SCI subpopulations most likely to benefit from experimental therapies as part of ongoing or future clinical trials.

Previous investigations of acute SCI biomarkers have demonstrated the utility of CSF and blood-based biomarkers for monitoring SCI. Elevated levels of NF-L and GFAP, both protein biomarkers of neuronal injury, measured in CSF and blood during the acute phase of SCI have been demonstrated to correlate with injury severity at presentation and 6-month clinical outcome (15). Given the relative ease of obtaining peripheral blood compared with CSF, establishing a blood biomarker for acute SCI would provide a more facile means of SCI assessment that may be implemented in a wider range of clinical contexts. Metabolic profiles of serum from acute SCI patients have been demonstrated to mirror those measured in parallel CSF samples (13). Similarly, changes in miRNA expression measured in serum reflect those measured in CSF from acute SCI patients (14). Moreover, studies that performed transcriptomic profiling of white blood cells in blood samples obtained in acute SCI patients demonstrated its potential to diagnose SCI, predict injury severity, and prognosticate clinical outcomes (24, 26). These findings suggest that blood biomarkers may provide comparable ability to CSF to detect molecular processes that occur following acute injury to inform diagnosis and prognosis.

We observed marked variation in the plasma cfDNA concentrations among SCI patients, including those with similar injury severity measured by ASIA score. cfDNA levels are known to vary based on patient age, disease status, and other factors (27, 28). This biological phenomenon challenges our ability to apply universal predetermined cutoffs to assess injury severity based on measured levels of spinal cord–derived cfDNA. Future studies will need to be conducted to correlate quantity of spinal cord–derived cfDNA and longitudinal assessment of injury severity.

cfDNA and protein measurements may provide orthogonal data for SCI assessment. Compared with cfDNA, circulating protein signatures may enable more sensitive detection of SCI (29). In our cohort, injured patients in whom spinal cord–derived cfDNA was not detected still demonstrated elevated levels of protein biomarkers. Plasma cfDNA and proteins may detect distinct molecular changes that occur during the acute phase of cell injury. cfDNA is understood to be a byproduct of apoptotic cell death. On the other hand, plasma proteins may indicate the occurrence of other cellular injury processes, such as mitochondrial dysfunction or neuronal membrane damage, as well as nonprogrammed cell death. We found that levels of spinal cord–derived cfDNA and selected plasma proteins were correlated with each other in injured patients, potentially due to their association with related cellular injury processes. Moreover, we tested the additive value of protein biomarkers and spinal cord–derived cfDNA for SCI diagnosis and prognostication. Integrating protein and cfDNA data into a composite score, the SCII, improved technical performance and resulted in potential predictive capacity for a clinically meaningful endpoint (6-month ASIA conversion). The performance of the SCII provides early evidence that a multi-analyte blood test for acute SCI may provide insight into long-term functional outcomes.

### Limitations and future directions.

There are several limitations of our study worth considering. Despite our array-based methylation profiling of spinal cord tissue to identify spinal cord–derived methylation patterns, similar methylation patterns may be shared by tissue types not specifically assessed. Broader epigenetic profiling (e.g., methylome wide) may provide additional targets for cfDNA analysis. To further improve the specificity of our methylation-based ddPCR approach for spinal cord–derived cfDNA, whole genome methylation sequencing of spinal cord tissue and more exhaustive comparison with other common tissue types will be required. Moreover, multiplex detection of several spinal cord–specific DNA targets may increase the overall sensitivity of a blood test for acute SCI.

In this initial version of a blood test for acute SCI, we also employed a proteomic panel that measured known protein markers of CNS disease. Discovery-oriented investigations of the plasma proteome may identify novel protein biomarkers of acute SCI that improve the performance of blood-based liquid biopsy. Additionally, RNA levels, though not measured in our investigation, may inform SCI assessment. Previously, microRNAs detected in CSF have been demonstrated to correlate with injury severity and clinical outcomes (14). Whether additional molecules, such as various RNA species and metabolites, measured in plasma can provide diagnostic and prognostic utility as part of a more comprehensive multi-analyte assay has yet to be determined.

Future investigations that apply the SCII to prospective independent patient cohorts are needed to validate its diagnostic and prognostic ability. Additionally, in order to characterize the ability of a multi-analyte blood test to capture dynamic neuroglial injury processes and response to treatment, future studies that obtain data at serial time points following decompressive surgery and other adjuvant therapies for spinal injury are needed. Characterizing the longitudinal and temporal dynamics of a multi-analyte assay that includes spinal cord–derived cfDNA may inform its potential integration into clinical trials as an exploratory companion diagnostic.

Finally, in our initial version of a multi-analyte blood test for acute SCI, rapid time from sample collection to data acquisition is limited by our methylation-based ddPCR approach for measuring spinal cord–derived cfDNA. For blood-based liquid biopsy to provide real-time assessment of patient injury and inform clinical decision making, methods that provide data on cfDNA methylation status within an approximate time frame of an hour are necessary. Methylation-sensitive loop-mediated isothermal amplification (LAMP) and mobile fluorescent readout devices may enable methylation detection at target DNA sites within an hour of DNA isolation (30). Additionally, advances in next-generation sequencing, such as targeted DNA methylation analysis via nanopore sequencing, may support faster turnaround times (31, 32). Ultimate application of a blood test for acute SCI in a clinical setting will require integrating the proof of concept illustrated by our initial methylation-based ddPCR approach with technologies that support point-of-care methylation measurement.

## Methods

### Sex as a biological variable.

Our study included male and female patients.

### Patient selection.

Patients at least 18 years of age who were diagnosed with acute SCI were prospectively recruited at Johns Hopkins Hospital and Bayview Medical Center. Diagnosis of acute SCI was based on patient report of new neurologic deficit and evidence of SCI on MRI. Included SCI etiologies were tSCI, traumatic CCS, and acute ESCC. Patients who presented beyond 7 days from new neurologic deficit were excluded. Patients who received blood product transfusions prior to preoperative blood sample procurement were also excluded. Patient samples (*n* = 50) were initially used for optimization of the ddPCR assay. A subset of patient samples was included for proteomic profiling (*n* = 34) based on sample availability after optimization of the ddPCR assay.

### Sample collection and processing.

Blood samples were obtained from arterial or venous access lines within 7 days of the initial injury and prior to surgical intervention. Between 10 and 30 mL of peripheral blood was collected in cfDNA blood collection tubes (Streck). Tubes were centrifuged for 10 minutes at 814*g* to separate the plasma. Plasma was transferred to a standard 15 mL tube and centrifuged a second time at 5,662*g* for 30 minutes. Supernatant containing cfDNA was stored at –80°C.

### Identification of spinal cord–specific CpG sites.

Genome-wide methylation analysis using Illumina MethylationEPIC 850K arrays was performed on fresh, frozen (*n* = 3), and FFPE human spinal cord tissue (*n* = 3) from control patients. DNA methylation analysis was performed using the *minfi* package in R, version 4.1.0 (The R Foundation for Statistical Computing) (33). An atlas of DNA methylation data for 25 different healthy tissue types was compiled from publicly available data (see Supplemental Table 2) (34). A beta value (β) was generated for each CpG site, where β = 0 indicated a completely unmethylated signal and β = 1 indicated a completely methylated signal. CpG sites that were differentially methylated between spinal cord tissue and other tissues were determined using the *dmpFinder* function in *minfi*. To measure differential methylation effect size, we computed the mean and median β value difference between spinal cord tissue and all other tissues. CpG sites that were significantly differentially methylated and that had large methylation difference effect sizes were selected as candidate CpG sites. Furthermore, CpG sites within 100–200 base pairs of one another were prioritized for downstream analysis.

### Primer/probe design for ddPCR.

Custom ddPCR primer/probe pairs were designed to target 2 chromosomal loci containing CpGs that were differentially hypomethylated in spinal cord tissue. Assay 1 targeted chromosome 16 and 2 CpG sites (cg27088725, cg07209034), with 1 probe covering each locus. Assay 2 targeted chromosome 12 and 3 CpG sites (cg24336338, cg03776878, cg23617848), with 1 probe covering 2 CpG sites and the other probe covering one CpG. With this approach, double positive fluorescence indicated that all target CpGs were unmethylated on a single cfDNA molecule (35).

### cfDNA extraction and bisulfite conversion.

Plasma from banked blood samples was removed from –80°C storage and thawed on ice. The BioChain cfPure Cell Free DNA Extraction Kit was used to extract cfDNA from plasma. Plasma was centrifuged at 5200*g* for 30 minutes at 4°C. Supernatant containing cfDNA was transferred for extraction. Supernatant containing cfDNA was enzymatically lysed to remove bound proteins and prepared with buffers to inhibit DNases. Solution was combined with magnetic beads to bind cfDNA. Beads were washed with washing buffers and ethanol. Nuclease-free water was used to elute cfDNA into a working solution. Concentration of cfDNA (ng/μL) was measured using Qubit. Purity of cfDNA was assessed using the Agilent 2100 Bioanalyzer system and High Sensitivity DNA Assay protocol. Bisulfite conversion of extracted cfDNA was performed using the EpiTect Plus DNA Bisulfite Kit (QIAGEN).

### ddPCR.

Reaction volumes of 20 μl, consisting of 19 μl mastermix (11 μl Supermix for probes [no deoxyuridine triphosphate], 7 μl of nuclease-free water, 1 μl of each primer/probe mix for both the Fam and Hex probes), and 1 μl cfDNA sample of patient plasma were prepared and used for droplet generation. Droplets were transferred to a 96-well plate for DNA amplification and thermal cycling. ddPCR was performed using the QX200 ddPCR system according to the manufacturer’s instructions (Bio-Rad Laboratories). QuantaSoft, version 1.7.4.0917 (Bio-Rad Laboratories), software was used for data analysis.

Prior to plasma sample testing, thermal gradient experiments were performed on gDNA extracted from spinal cord tissue to determine optimal amplification conditions for thermal cycling. Based on clearest separation of negative and positive droplet clusters, thermal cycling conditions were set at 95°C for 10 minutes (1 cycle), 94°C for 30 seconds, 55°C for 60 seconds (40 cycles), and infinite hold at 12°C. For quality control, wells with total droplet counts of less than 10,000 were considered invalid and excluded from analysis. Samples containing gDNA from human spinal cord tissue were used to verify assay performance. Thresholds in fluorescence values were determined using negative control wells containing gDNA from human leukocytes and positive control wells containing gDNA from human spinal cord tissue.

Thresholds were applied to ddPCR reads of patient plasma samples to determine the number of double-positive droplets. Unique plasma samples were analyzed in triplicate. Estimated target DNA concentrations (copies/μl) were calculated using the formula *C* = −ln(*N_neg_*/*N*_tot_)/*V*_droplet,_ such that *C* = sample concentration (copies/μl), *N_neg_* = number of negative droplets, *N_tot_* = total number of droplets, *V_droplet_* = volume of droplet (1 nl). Plasma concentration in hGE/mL was calculated using the formula *PC* = *C* × *RV* × (*EV*/*TV*)/*PV*, such that *PC* = plasma concentration (copies/ml), *C* = sample concentration (copies/μl), *RV* = PCR reaction volume (20 μl), *EV* = volume in which cfDNA was eluted (15 μl); *TV* = volume of cfDNA added to the PCR reaction (1 μl), and *PV* = volume of plasma used for cfDNA extraction (5 ml). Conversion factor 1 ng = 303 hGE of cfDNA was used to calculate the plasma concentration of spinal cord–derived cfDNA in ng/mL. Percentage of spinal cord–derived cfDNA was calculated by dividing the plasma concentration of spinal cord–derived cfDNA (ng/mL) over the total plasma concentration of cfDNA measured via Qubit prior to bisulfite conversion.

### Proteomic profiling.

The NULISA 119-plex neurologic panel is designed to quantify proteins involved in neurological diseases and can achieve attomolar level detection. Plasma volumes of 50 μL were subjected to analysis via the NULISA proteomic platform. Briefly, this platform employs a unique dual-DNA barcode strategy, wherein capture Abs are tagged with double-stranded DNA featuring a poly(A) tail and barcode, and detection Abs are tagged with a complementary biotinylated barcode. The presence of the target protein prompts the formation of an immunocomplex. This complex is then isolated using paramagnetic oligo(dT) beads, leveraging dT-poly(A) hybridization. A wash process in a salt-sensitive environment subsequently releases these complexes into a low-salt buffer. Streptavidin-coated beads are then introduced, effecting a secondary capture phase for the complexes and eliminating nonspecifically bound Abs through further washing, culminating in the isolation of near-pure immunocomplexes. In the final step, the binding of a specialized DNA ligation sequence via T4 DNA ligase creates a DNA reporter molecule that embodies the unique target-specific barcodes. These reporters are quantitatively analyzed by next-generation sequencing. Data normalization — achieved using internal controls — addresses technical variability, and a rescaling and log_2_ transformation process yields the final NPQ units.

### Derivation of the SCII.

Proteins were ranked based on difference in median NPQ values between SCI patients who achieved 6-month ASIA conversion and those who did not, in a post hoc fashion. An initial inclusion threshold was set by assessing the distribution of these differences. Proteins with a median difference of 2 or more standard deviations above the mean difference were included in the forward selection process. Forward selection in a logistic regression model was used to identify a parsimonious set of proteins associated with 6-month ASIA conversion. Proteins were sequentially added to the model, starting with the highest ranked and continuing until the model converged. The coefficients of selected proteins were linearly combined with log_10_ scaled spinal cord–derived cfDNA concentration to define a composite SCII. The same methodology was used, but comparing SCI patients with non-SCI controls, to define a composite score specifically aimed for SCI diagnosis. This identified 5 proteins (NF-H, NF-L, IL-10, serum amyloid A1 [SAA1], and S100 calcium-binding protein A12 [S100A12]) that were linearly combined with a log_10_ scaled spinal cord–derived cfDNA concentration. As depicted in Supplemental Figure 5, this approach expectedly achieves robust performance for discriminating between SCI patients and non-SCI controls (AUC, 0.95) but demonstrated suboptimal association with injury severity and prediction of 6-month ASIA conversion (AUC, 0.57).

### Statistics.

Significant differences between groups were determined using Mann-Whitney *U* tests. *P* values were considered significant when less than 0.05. Modeling was performed in the R statistical language and visualization formed using GraphPad Prism, version 10.3.1.

### Study approval.

This study was approved by the Johns Hopkins University IRB Board under protocols IRB00237129 and IRB00290670. Informed consent was obtained from patients or legally authorized representatives prior to study participation.

### Data availability.

Values for all data points in graphs are reported in the Supporting Data Values file. Patient level data is available from the corresponding author upon reasonable request and IRB approval.

## Author contributions

Shared first authorship was decided based on respective contributions to study design, method development, and experimental execution. TDA, NT, and CB conceived the project. TDA, TAK, SY, and CB designed methodology. TDA, KRR, JDM, DR, TAK, ML, SG, ETW, AKA, MP, ALK, DL, CMJ, DMS, JDW, AB, TFW, and DWN conducted investigations. TDA, KRR, SG performed visualization. TDA, NT, and CB acquired funding. TDA, NT, and CB supervised project administration. TDA, CB, and NT supervised the project. TDA KRR wrote the original draft of the manuscript. All authors reviewed and edited the manuscript.

## Supplementary Material

Supplemental data

ICMJE disclosure forms

Supporting data values

## Version 1 03/03/2025

Electronic publication
