# Supplementary material for: A multi-analyte blood test for acute spinal cord injury
Source: J Clin Invest. 2025 Mar 3;135(5):e185463. doi: 10.1172/JCI185463 (PMC11870727; doi:10.1172/JCI185463)
Supplement: Supplemental data [file jci-135-185463-s246.pdf]

List of Supplemental Materials

Tables: S1-S2

Figures: S1-S5

**Characteristic**	N (%)
**SCI patients**	50
Age (yrs), mean ± SD	60.1 ± 14.9
Sex (male)	34 (68.0)
Injury type	
ESCC	17 (34.0)
CCS	19 (38.0)
tSCI	14 (28.0)
Preoperative ASIA	
A	6 (12.0)
B	12 (24.0)
C	13 (26.0)
D	19 (38.0)
Level of injury	
Cervical	30 (60.0)
Thoracic	20 (40.0)
Surgical approach	
Anterior	3 (6.0)
Posterior	46 (92.0)
**Control subjects**	20
Age (yrs), mean ± SD	30.8 ± 9.9
Sex (male)	11 (55.0)

Supplemental Table 1. Overview of study cohort, including patients with acute spinal cord injury and control subjects.

SD – standard deviation; ESCC – epidural spinal cord compression; CCS – central cord syndrome; tSCI – traumatic spinal cord injury; ASIA – American Spinal Injury Association Impairment Scale

Tissue	GEO ID
Stomach	GSE99553
Breast	GSE124367
Cervix	GSE135446
Myometrium	GSE135446
Esophagus	GSE72872
Prostate	GSE112047
RBC progenitors	GSE63409
Chondrocytes	GSE63695
Left atrium	GSE62727
Muscle	GSE151407
Thyroid	GSE146003
Adipocytes	GSE122126
Colon epithelium	GSE122126
Endothelium	GSE122126
Hepatocytes	GSE122126
Lung epithelium	GSE122126
Pancreatic acinar cells	GSE122126
Pancreatic beta cells	GSE122126
Pancreatic ductal cells	GSE122126
B cells	GSE110554
$CD4^+$ T cells	GSE110554
$CD8^+$ T cells	GSE110554
Monocytes	GSE110554
Neutrophils	GSE110554
NK cells	GSE110554

Supplemental Table 2. References for DNA methylation tissue atlas.

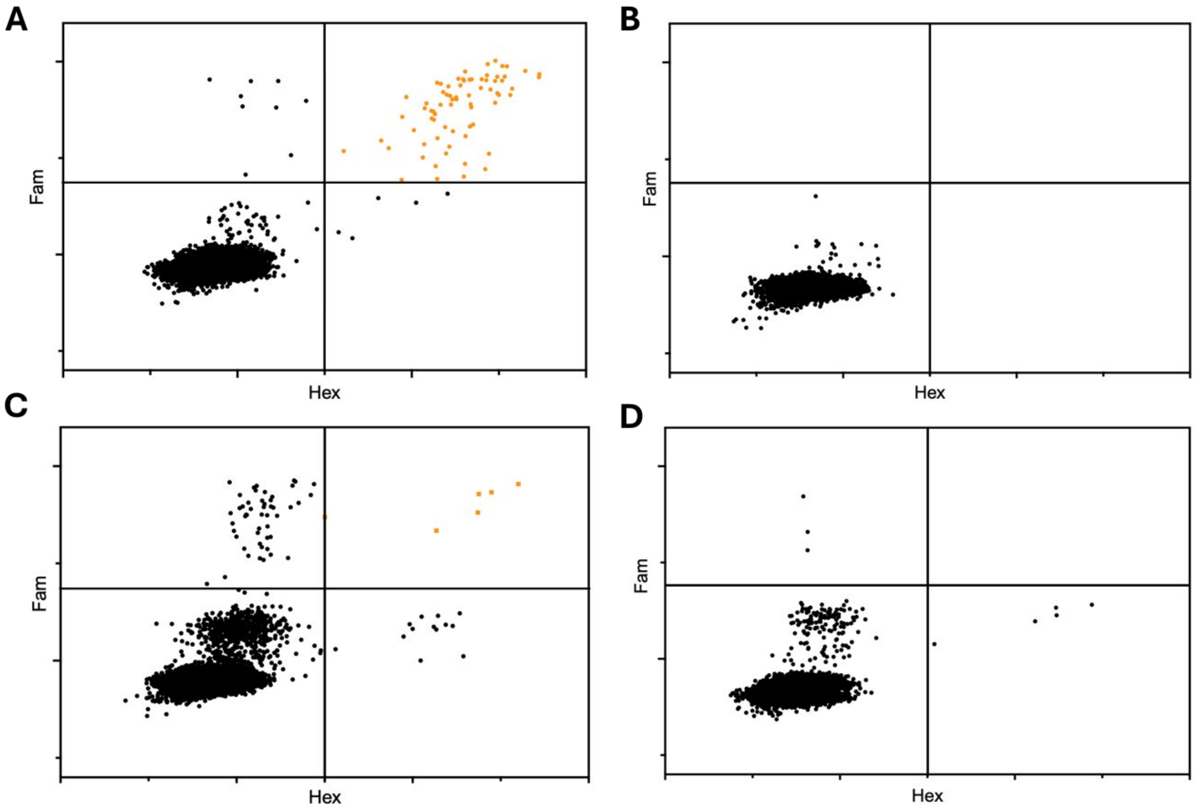

Supplemental Figure 1. Representative 2D ddPCR plots for (**A**) spinal cord genomic DNA extracted from healthy patient rapid autopsy spinal cord tissue (**B**) cell-free DNA extracted from healthy patient plasma (**C**) cell-free DNA extracted from acute SCI patient plasma and (**D**) genomic DNA extracted from acute SCI patient peripheral blood mononuclear cells.

DdPCR – digital droplet PCR; SCI – spinal cord injury

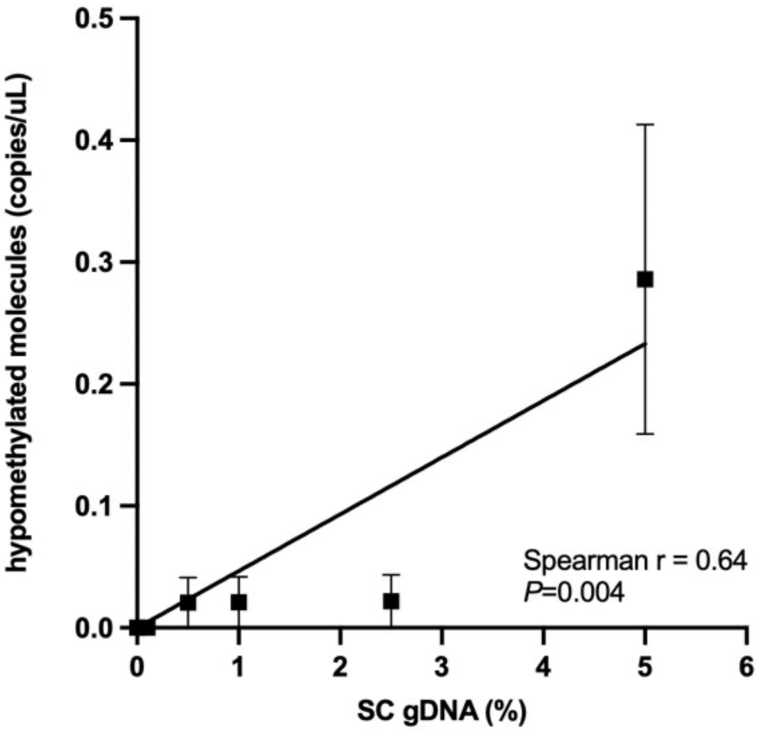

Supplemental Figure 2. Spike-in experiment using 3 CpG site assay demonstrates poor technical calibration for detection of spinal cord neuron-specific biomarkers. Human spinal cord gDNA was mixed with human leukocyte gDNA in the indicated proportions (0 to 5%), and the concentration (copies/uL) of fully unmethylated spinal cord neuron markers was determined. Data represent mean ± SEM.

SC – spinal cord; gDNA – genomic DNA; SEM – standard error of measurement

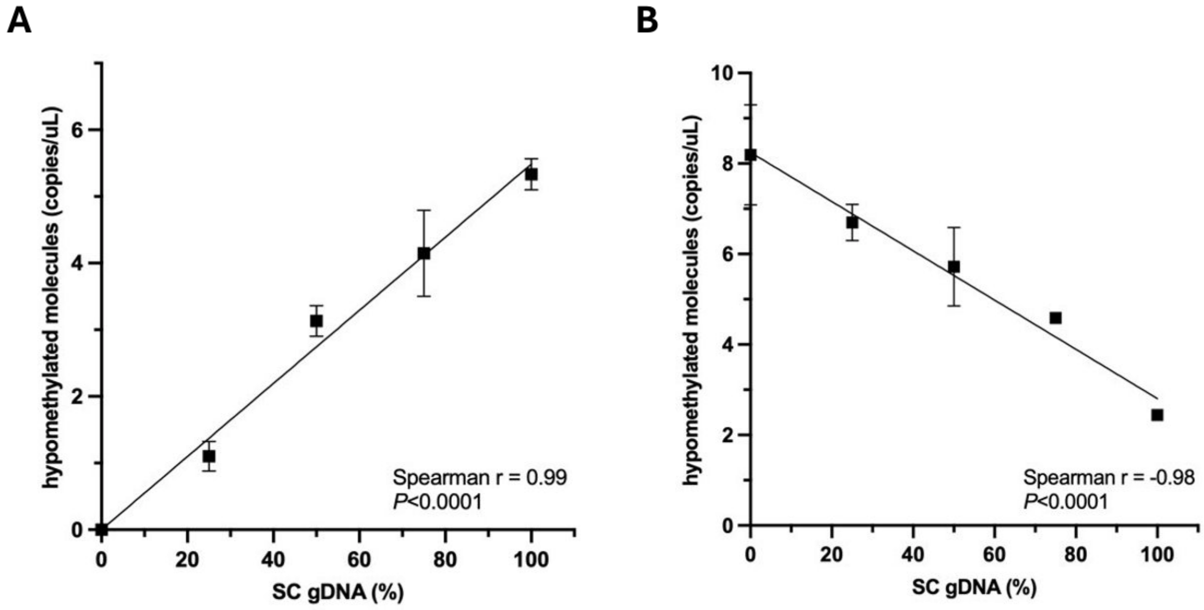

Supplemental Figure 3. Spike-in experiment using Assay 1 (**A**) and reversed Assay 1 design (**B**). Human spinal cord gDNA was mixed with human leukocyte gDNA in the indicated proportions and the concentration (copies/uL) of unmethylated DNA (**A**) or methylated DNA (**B**) was determined. Data represent mean ± SD.

SC – spinal cord; gDNA – genomic DNA; SD – standard deviation

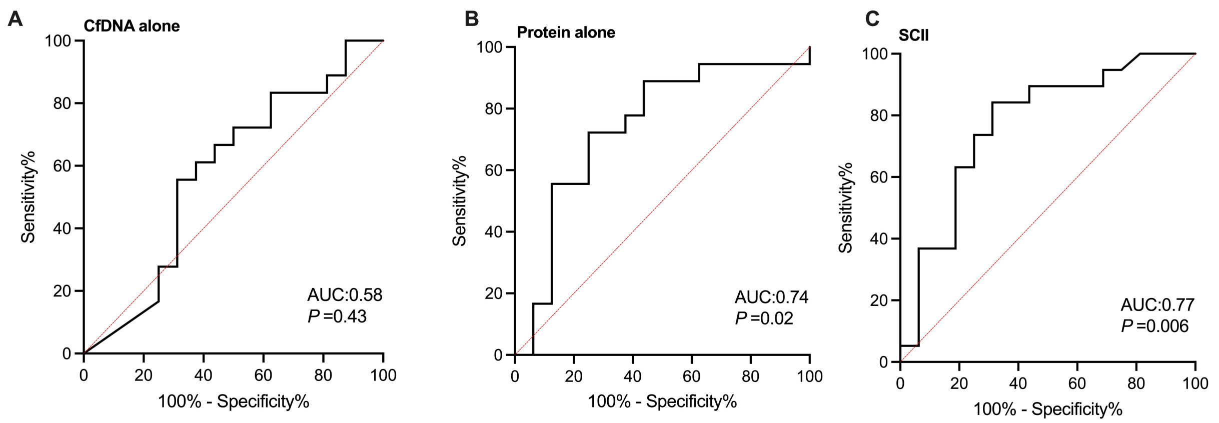

Supplemental Figure 4. Performance of cfDNA alone, protein alone, and composite Spinal Cord Injury Index (SCII) for discrimination of 6-month ASIA conversion. (**A**) Receiver Operating Characteristic (ROC) curve for ability of spinal cord-derived cfDNA to predict 6-month ASIA conversion. (**B**) ROC curve for ability of protein biomarkers to predict 6-month ASIA conversion. (**C**) ROC curve for ability of composite SCII to predict 6-month ASIA conversion.

cfDNA – cell-free DNA; SCII – Spinal Cord Injury Index; ROC – receiver operating characteristic; ASIA - American Spinal Injury Association Impairment Scale

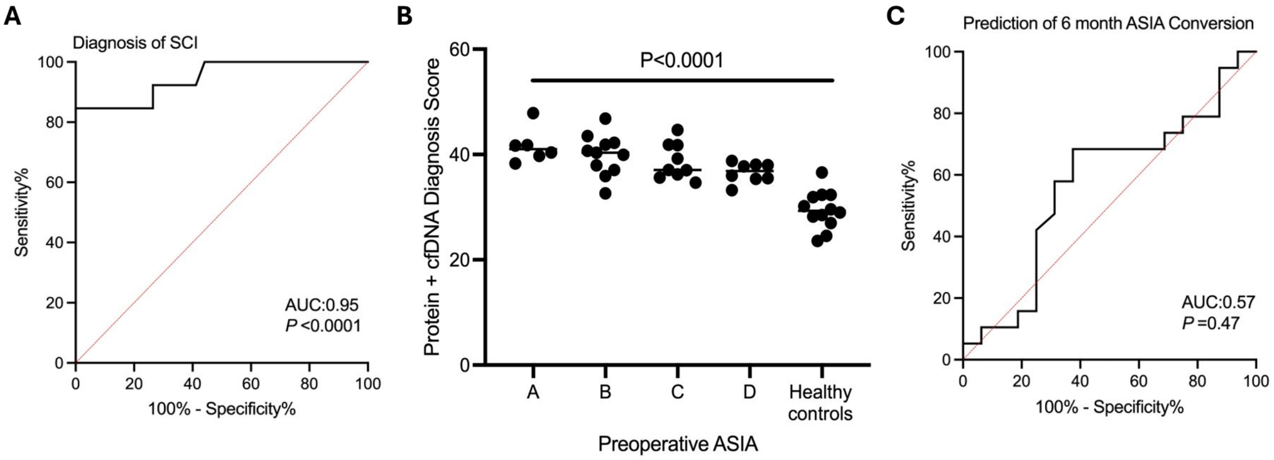

Supplemental Figure 5. Performance of integrated protein and spinal cord-specific cfDNA ("diagnosis") score tuned for acute SCI versus healthy control discrimination. Included proteins: NF-H, SAA1, S100A12, IL-10, NF-L. (**A**) Receiver Operating Characteristic (ROC) curve for ability of diagnosis score to discriminate between patients with acute SCI and healthy controls. (**B**) Distribution of diagnosis score across injury severity. (**C**) ROC curve for ability of preoperative diagnosis score to predict 6-month ASIA conversion.

SCI – spinal cord injury; cfDNA – cell-free DNA; NF-H – neurofilament, heavy polypeptide; IL-10 – interleukin 10; SAA1 – serum amyloid A1; S100A12 - S100 calcium-binding protein A12; NF-L – neurofilament light; ROC – receiver operating characteristic; ASIA – American Spinal Injury Association Impairment Scale
